# Spatio-Temporal Variation of PM_2.5_ Concentrations and Their Relationship with Geographic and Socioeconomic Factors in China

**DOI:** 10.3390/ijerph110100173

**Published:** 2013-12-20

**Authors:** Gang Lin, Jingying Fu, Dong Jiang, Wensheng Hu, Donglin Dong, Yaohuan Huang, Mingdong Zhao

**Affiliations:** 1 College of Geoscience and Surveying Engineering, China University of Mining & Technology, Ding No.11 Xueyuan Road, Haidian District, Beijing 100083, China; E-Mails: ling@lreis.ac.cn (G.L.); ddl@cumtb.edu.cn (D.D.); zhaomd@lreis.ac.cn (M.Z.); 2 State Key Laboratory of Resources and Environmental Information System, Institute of Geographical Sciences and Natural Resources Research, Chinese Academy of Sciences, 11A Datun Road, Chaoyang District, Beijing 100101, China; E-Mails: fujingying.2005@163.com (J.F.); huangyh@lreis.ac.cn (Y.H.); 3 University of Chinese Academy of Sciences, No. 19 Yuquan Road, Beijing 100049, China

**Keywords:** PM_2.5_, GDP, population, land use change, geographically weighted regression

## Abstract

The air quality in China, particularly the PM_2.5_ (particles less than 2.5 μm in aerodynamic diameter) level, has become an increasing public concern because of its relation to health risks. The distribution of PM_2.5_ concentrations has a close relationship with multiple geographic and socioeconomic factors, but the lack of reliable data has been the main obstacle to studying this topic. Based on the newly published Annual Average PM_2.5_ gridded data, together with land use data, gridded population data and Gross Domestic Product (GDP) data, this paper explored the spatial-temporal characteristics of PM_2.5_ concentrations and the factors impacting those concentrations in China for the years of 2001–2010. The contributions of urban areas, high population and economic development to PM_2.5_ concentrations were analyzed using the Geographically Weighted Regression (GWR) model. The results indicated that the spatial pattern of PM_2.5_ concentrations in China remained stable during the period 2001–2010; high concentrations of PM_2.5_ are mostly found in regions with high populations and rapid urban expansion, including the Beijing-Tianjin-Hebei region in North China, East China (including the Shandong, Anhui and Jiangsu provinces) and Henan province. Increasing populations, local economic growth and urban expansion are the three main driving forces impacting PM_2.5_ concentrations.

## 1. Introduction

Fine particulate matter (PM_2.5_, *i.e.*, particles less than 2.5 μm in aerodynamic diameter) is rich in organic toxic components and has a strong association with many adverse health effects [1,2,3]. Epidemiological studies have reported associations between PM_2.5_ and a variety of medical diseases such as asthma, cardiovascular problems, respiratory infections, lung cancer [4] and breast cancer. The air quality in China, particularly the PM_2.5_ level, has become an increasing public concern because of its connection to such health risks. For example, Hu suggested that exposure to high PM levels may have deleterious effects on the duration of survival after a breast cancer diagnosis among females [5]. Zhang *et al.* estimated the number of people living in high-exposure areas in Beijing during the autumn of 2012 [6].

Accurate modeling of fine scale spatial variation in PM_2.5_ concentrations is critical for environmental and epidemiological studies. Land use regression (LUR) models are widely employed to expand *in situ* measurements of PM_2.5_ concentrations to large areas. LUR is essentially an interpolation technique that employs the PM_2.5_ concentrations as the dependent variable, with proximate land use, traffic and physical environmental variables used as independent predictors [7,8]. Some literature has suggested that PM emissions in urban areas has come from road traffic, household activities, energy production, building work, (inland) shipping and (small-scale) industry [9]. However, the lack of long-term monitoring data is the primary obstacle in developing countries where PM_2.5_ concentration sites are sparse and have only been established in recent years. Fortunately, recent studies have indicated that satellite-observed total-column aerosol optical depth (AOD) could offer spatially continuous information about PM_2.5_ concentrations at the global scale [10]. Mao *et al.* developed an AOD-enhanced space-time LUR model to predict the PM_2.5_ concentrations in the state of Florida in the USA [11]. Van *et al.* presented a global estimate of PM_2.5_ concentrations at 10 km × 10 km resolution for six years (2001–2006) by combing satellite-derived AOD with *in situ* measurements [12]. In June 2013, the Global Annual PM_2.5_ Grids were published by Battelle Memorial Institute and the Center for International Earth Science Information Network (CIESIN)/Columbia University; this data set represents a series of annual average grids (2001–2010) [13]. The global, 0.5 × 0.5 grid of estimated PM_2.5_ concentrations was developed using monthly AOD data from MODIS and MISR for the period 2001–2010; these estimates leveraged the AOD/PM_2.5_ surface level conversion factors calculated by van Donkelaar [12] and applied them to the gridded remote sensing data. The gridded product provides a continuous surface of PM_2.5_ concentrations in micrograms per cubic meter for health and environmental research.

The objective of this paper is to explore the spatio-temporal characteristics and driving forces of PM_2.5_ concentrations in China based on long-term newly refined data. Annual Average PM_2.5_ gridded data, land use data, gridded population data and Gross Domestic Product (GDP) data for the period 2001–2010 were used in the analysis. The contributions of urban areas, high population and economic development to PM_2.5_ concentrations were analyzed using the Geographically Weighted Regression (GWR) model. 

## 2. Data Acquisition

### 2.1. PM_2.5_ Data

The Global Annual Average PM_2.5_ Grids represent a series of annual average grids (2001–2010) of PM_2.5_, these data were obtained from the Battelle Memorial Institute and the Center for International Earth Science Information Network (CIESIN)/Columbia University, and each file obtained from Battelle/CIESIN contains integer values for a global, 0.5 × 0.5 grid of estimated PM_2.5_ concentrations. The average annual PM_2.5_ concentration for each grid cell was calculated by multiplying the MODIS and MISR mean AOD for each month by the monthly conversion factor, as in Equation (1) [13]:

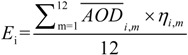
(1)
where *E_i_* stands for an annual-average estimated PM_2.5_ concentration for each grid cell; *AOD_i_*,*_m_* stands for the MODIS and MISR mean AOD for each month; and *η_i_*_,*m .*_stands for the monthly conversion factor.

The PM_2.5_ dataset offers spatially continuous information about PM_2.5_ concentrations at the global scale while it also has some uncertainties. PM_2.5_ concentrations derived from Battelle/CIESIN has biased values, and they may be higher or lower than those for van Donkelaar *et al.* in some regions such as largely arid and semi-arid countries with large desert areas [13]. The reasons for these differences are unclear. Uncertainties or limitations of the AOD data and computing methods or some other possible reasons all can cause these biases.

**Figure 1:**
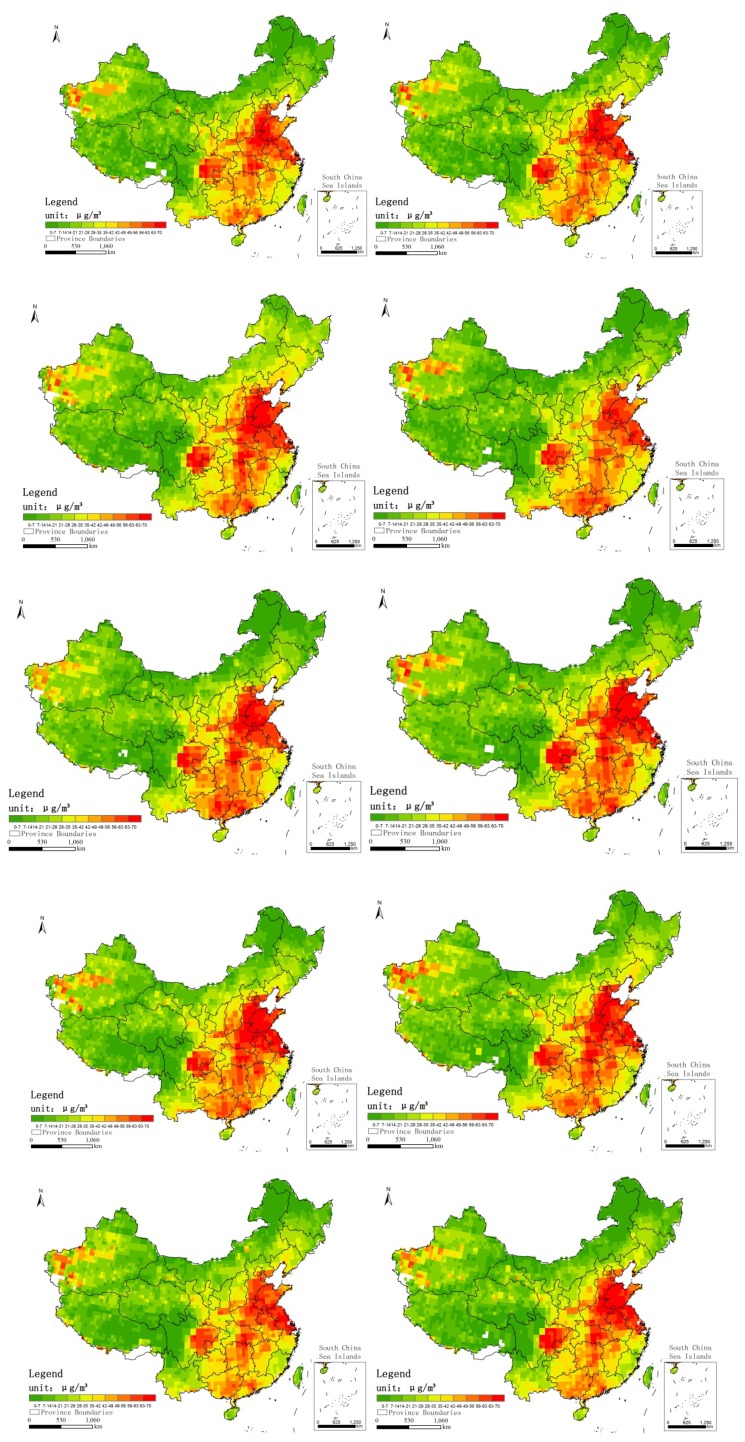
The estimated distribution of PM_2.5_ concentrations in China from 2001 to 2010.

Data for China were extracted from the global dataset using the ArcGIS software and were transformed to the same coordinate system as the other datasets, specifically the Albers Equal Area projection system, Beijing 1954 geodetic datum and Krassovsky ellipsoid. Figure 1 shows the estimated distribution of PM_2.5_ concentrations in China from 2001 to 2010.

### 2.2. Population Data

Gridded population data in China with a spatial resolution of 1km from 2001 to 2010 were provided by the Resources and Environmental Scientific Data Center (RESDC), Chinese Academy of Sciences (CAS) [14]. These gridded population data were transformed from census data based on the relationship between demographical data and land use types, and the population data were redistributed onto 1 km × 1 km grids [14].

### 2.3. GDP Data

GDP is a commonly used indicator of economic development. The gridded GDP dataset for China from 2001 to 2010 was adopted in this study. The dataset was obtained from RESDC, CAS. The statistical GDP data at the county level were transformed into gridded data at a resolution of 1 km × 1 km based on the relationship between the GDP data and the land use types [15].

### 2.4. Land-use Data

Land-use data for China at a scale of 1:100,000 for the years 2001 and 2010 were used in this study. The datasets were obtained using Landsat TM (Thematic Mapper) and the China-Brazil Earth Resources satellite (CBERS-2) satellite images and were interpreted by experts at RESDC, Chinese Academy of Sciences. The following six land use types were identified: (1) cultivated land; (2) woodland; (3) grass land; (4) water; (5) urban and rural settlements; and (6) barren land. Areas of urban sprawl were derived from the land use data. A set of land data from field surveys was selected to guarantee the accuracy of land use classification and it is the most accurate land use dataset at this scale in China [16]. Before further processing, all of the source data were re-sampled onto a raster dataset with 1 km spatial resolution, and transformed into the same coordinate system.

## 3. Methodology

The spatio-temporal variations of the PM_2.5_ concentrations and their relationships with socioeconomic factors were evaluated using the following steps:

Step 1: Evaluate the spatio-temporal variation of PM_2.5_ concentrations in China from 2001 to 2010 based on annual average PM_2.5_ grids.
Step 2: Compare the distribution of PM_2.5_ concentrations with each of the following factors: urban areas, population and GDP. The impact of each factor on the PM_2.5_ concentrations was analyzed and compared.
Step 3: Use the GWR method to evaluate the relationships between the PM_2.5_ concentrations and the urban areas, population and GDP.

A conventional regression method, such as ordinary least squares (OLS), is a type of global statistic that assumes that the relationship under study is constant over space and therefore assumes that the parameter is the same for the entire study area [17]. The GWR model extends the traditional standard regression framework to estimate local, rather than global, parameters [18]. The GWR model is a type of local statistic that produces a set of local parameter estimates that show how a relationship varies over space. This visualization enables examining the spatial pattern of the local statistics to gain a better understanding of possible hidden causes for that pattern [19]. The global regression model can be expressed as follows:

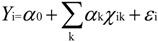
(2)

We can obtain the vector estimates of the parameters using OLS:

*α* = (*X^T^ X*)^‒1^*X^T^Y* (3)

Where α is the vector estimate of the parameters, and X is the matrix composed of the observed values of the independent variable; the first column elements of X are 1. Y is composed of the observed values of the dependent variable [20].

The GWR method considered the local estimates of the parameters, and the model is extended to (1):

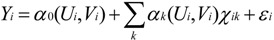
(4)
Where (*U_i_*,*V_i_*) is the space coordinate of sample point *i* and *α_k_*(*U_i_*,*V_i_*) is the value of the continuous function *α_k_*(*U*,*V*) on point *i*. If *α_k_*(*U*,*V*) remains unchanged in space, the model (3) is translated into the global regression model. Therefore, the GWR equation considers the spatial variability of the relationship:

*A*(*U_i_*,*V_i_*) = (*X^T^W* (*U_i_*,*V_i_*)*X*)^‒1^*X^T^ W*(*U_i_*,*V_i_*)*Y* (5)

where *W*(*U_i_*,*V_i_*) is the range weight matrix [20,21].

## 4. Results and Analysis

### 4.1. Spatio-temporal Variation of PM_2.5_ Concentrations in China

**Figure 2:**
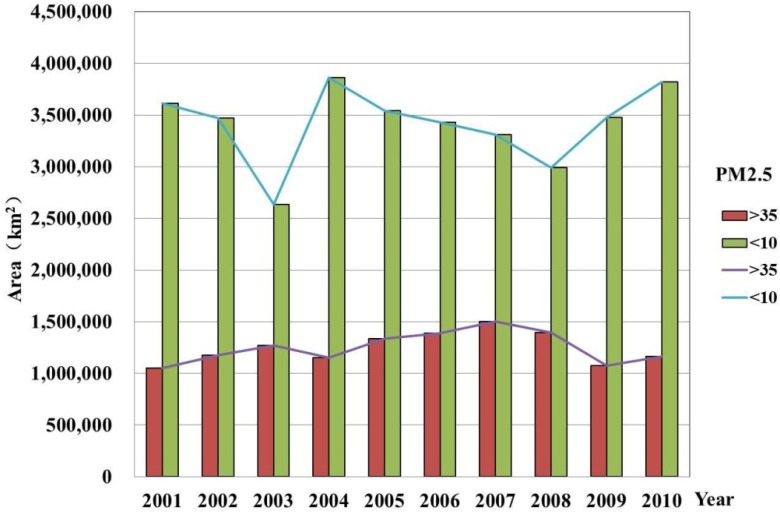
Temporal profiles of the areas in China for which PM_2.5_ concentrations are below 10 μg/m^3^ or above 35 μg/m^3^ from 2001 to 2010.

The World Health Organization (WHO) defined the standard for the annual average PM_2.5_ concentration to be less than 10 μg/m^3^ [22]. The human illness rate will increase immensely when the annual average concentrations reach 35 μg/m^3^ (Target 1 level). However, China’s air quality standard for the annual average PM_2.5_ concentration is 35 μg/m^3^ [23]. Temporal profiles of the areas of each PM_2.5_ concentration level from 2001 to 2010 are shown in Figure 2.

**Figure 3:**
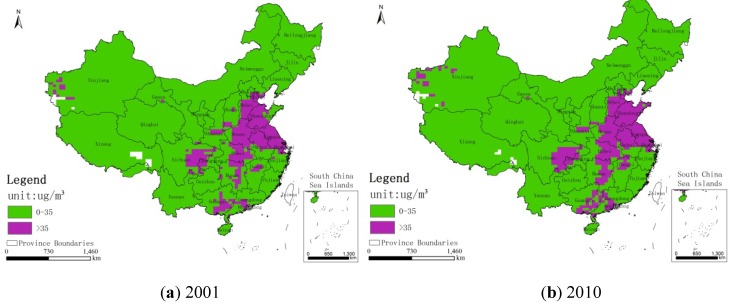
WHO target levels for PM_2.5_ concentrations in regions of China for (**a**) 2001 and (**b**) 2010.

**Figure 4:**
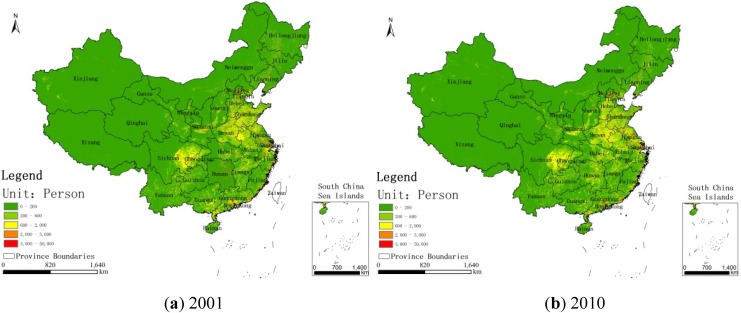
Population distribution in China for (**a**) 2001 and (**b**) 2010.

**Figure 5:**
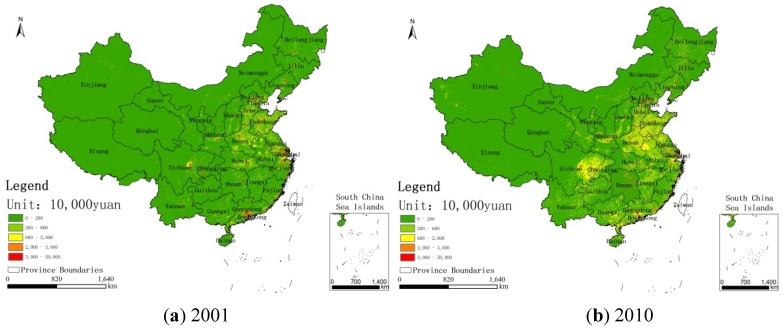
GDP distribution in China for **(a)** 2001 and **(b)** 2010.

**Figure 6:**
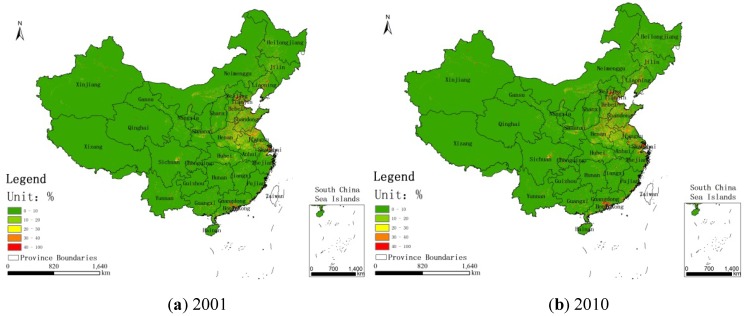
Urban areas in China for (**a**) 2001 and (**b**) 2010.

Figure 3 shows the regions whose concentrations have exceeded 35 μg/m^3^ in China for the years 2001 and 2010. Figure 4, Figure 5, Figure 6 show the distribution of population, GDP and urban area in China for the years 2001 and 2010. From these maps, we can see the spatial variation of PM_2.5_, population, GDP and urban areas in China for 2001 and 2010 and the area of the IT-1 level defined by WHO (annual mean PM_2.5_ concentration in excess of 35µg/m^3^) slowly increased by 7.2%/a on average from 2001 to 2007 and decreased by 7.5%/a on average from 2007 to 2010.

We hypothesize that higher populations and GDP levels may cause higher PM_2.5_ concentrations and that a larger urban area results in higher PM_2.5_ concentrations. Thus, we build the regression model to study the correlation between the PM_2.5_ concentrations and the population, GDP and urban area in China for 2001 and 2010 to evaluate the determinants of the increase in PM_2.5_ concentrations. 

### 4.2. Correlation between PM_2.5_ Concentrations and Socioeconomic Issues

Before geographically weighted regression can occur, one initial statistical analysis can determine the characteristics of each of the variables proposed for the model. We use liner regression models to examine the correlation between PM_2.5_ and each variable. The summary statistical results are shown in Table 1. All of the associations with PM_2.5_ are in the expected direction.

**Table 1:** The summarized statistical results of the initial statistical analysis.

Variable		2001			2010	
		R *	Correlation		R *	Correlation
PM_2.5_	Population	0.41	positive		0.50	positive
	GDP	0.59	positive		0.58	positive
	Urban area	0.59	positive		0.59	positive

Notes: * R is the correlation coefficient between PM_2.5_ and population, GDP and urban area. All the results have statistical significance. The *P* value of each regression is less than 0.05.

We adopted the Variance Inflation Factor (VIF) to detect whether there existed the co-linearity problem among the indicators. With SPSS software, we found that the VIF values of population, GDP and urban area are 2.79, 2.58, 2.12 of the year 2001, separately; and 3.63, 3.60, 2.31 of the year 2010, separately. So we should not worry about the co-linearity problem since the VIF values range from 0 to 10, even though there may be some correlations among the three factors.

The GWR model is run with ArcGIS 9.3 software. We built a GWR model of the correlation between PM_2.5_ concentrations and population, GDP and urban area in China for 2001 and 2010. The GWR model produces a set of local regression results including local parameter estimates and local residuals, which can be mapped to show their spatial variability. In this study, we have chosen an ADAPTIVE kernel whose bandwidth will be found by minimizing the corrected Akaike Information Criterion (AICc) value.

**Figure 7:**
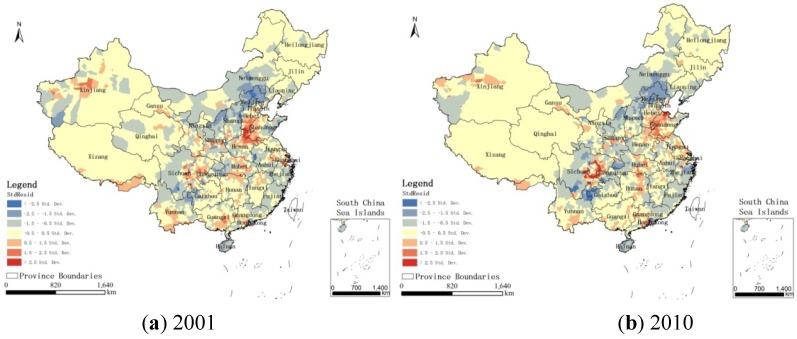
Maps of standardized residuals from the GWR model in China for the years (**a**) 2001 and (**b**) 2010.

From the report created by the GWR tool, we can obtain the local R^2^ and the local R^2^ Adjusted (the adjusted R^2^). The local R^2^ values for 2001 and 2010 are 0.820 and 0.822, respectively. The local R^2^ adjusted values for 2001 and 2010 are 0.810 and 0.815, respectively. The values for the two years are very closewhich can denote that the overall performance of the model relatively high in both of the two models. The values of the standardized residual (StdResid) for 2001 and 2010 can be mapped; these are shown in Figure 7. Not surprisingly, some unusually high or low residuals can be observed. Those regions with some desert area have very large residuals (StdResid > 2). For example, the PM_2.5_ concentrations in the northwest region of Xinjiang are high because of the desert. Xinjiang has a high incidence zone of dust explosion. The concentrations of dust aerosol at altitude are closely related to the surface conditions below; the concentrations of particles above the desert areas will be greater than those in the vegetation-covered areas [24]. Therefore, the high PM_2.5_ concentrations in desert regions are mainly related to the dusty weather. The southern Hebei province, the northern Henan province and the northwest Shandong province also have much higher residuals because they are high pollution emission regions of northern China. For example, the Shijiazhuang Iron and Steel Company discharges an average of more than 2000 t/a of PM_2.5_. There are also many polluting enterprises in the urban areas [25]. The pollution in these regions is always more serious than the pollution in other regions. In addition, the Sichuan basin has high residuals because of its high aerosol optical depth values. The optical depth of the Sichuan Basin is higher than its surrounding areas due to its geographical climate characteristics; its annual average optical depth is approximately 0.7 [26]. PM_2.5_ has a strong positive correlation with AOD [27], so the Sichuan Basin has high PM_2.5_ concentrations. Regions that are rich in marine salt can also have high PM_2.5_ concentrations [28]. Those regions have a noticeable over-prediction of PM_2.5_ concentrations; this warrants closer inspection to discover the possible explanations. In those regions, the model under-predicts the levels of PM_2.5_ concentrations [18]. However, the regions with StdResid values in the range of −2 to 2 account for 94.8% and 94.6% of the whole country, which indicates that the relations between PM_2.5_ and that each of the three factors are stable. What’s more, we can also obtain from the results that the regions with the positive value of the local coefficients for urban areas, population, and GDP account for 92.72%, 90.52% and 95.62% respectively in 2001 and 92.01%, 95.29% and 90.50% respectively in 2010 of the whole country. There is agreement with our expectation on the direction of the influence of those variables.

## 5. Discussion

Most of the associations between the PM_2.5_ concentrations and the other variables considered are in the expected direction. PM_2.5_ is correlated to population, GDP and urban area. Therefore, we have sufficient reason to believe that the regions with large populations, high values of GDP and large urban areas would have high values of PM_2.5_.

In China, PM_2.5_ mainly comes from human activities (motor vehicle tail gas dust and coal dust) and the karaburan; the ground dust and secondary particles of the karaburan also contribute to the PM_2.5_ concentrations [29,30]. Human activities that have a strong impact on the air quality are often sources of PM_2.5_; therefore, some cities with poor air quality have a high level of PM_2.5_. Metropolitan areas have large populations, high GDP values, large proportions of urbanization and industries that produce contaminants [31]. From Figure 2, we can see that the high PM_2.5_ values are mainly concentrated in the regions with large populations, high GDP values and large proportions of urbanization. For example, in Beijing, the values of PM_2.5_ have a linear relation with motor vehicle tail gas dust, coal dust and karaburan [32,33,34]. Population growth and economic development are accelerating the environmental deterioration in Beijing [31]. In addition, some research shows that, in Tianjin and Chongqing, the PM_2.5_ concentrations are dependent on motor vehicle exhaust dust, coal dust and karaburan [35,36,37]. In some large cities, the pollution from coal, other fuels and industrial pollution impacts particulate matter concentrations [38]. In addition, PM_2.5_ concentrations are dependent on temperature, humidity and rainfall in some regions [39]. Each area has its own leading factor that influences PM_2.5_ concentrations. Future work might include partitioning the different factors impacting PM_2.5_ concentrations.

## 6. Conclusions

In this study, spatio-temporal characteristics and factors impacting PM_2.5_ concentrations in China for the years 2001–2010 were evaluated based on newly refined long-term data. The following main conclusions are reached:

(1) In general, the spatial pattern of PM_2.5_ concentrations in China has remained stable during the period 2001–2010. The area of the IT-1 level defined by the WHO (annual mean PM_2.5_ concentration in excess of 35 µg/m^3^) slowly increased by 7.2%/a on average from 2001 to 2007 and decreased by 7.5%/a on average from 2007 to 2010.
(2) PM_2.5_ is mostly concentrated in regions with high populations, GDP and large urban regions, including the Beijing-Tianjin-Hebei region in north China, east China (including the Shandong, Anhui and Jiangsu provinces), the Henan province. The Sichuan basin is one exception to this result.

This paper, for the first time, presents a comprehensive insight into the spatio-temporal characteristics of PM_2.5_ concentrations in China at national scale. However, the problem is complex and needs further attention. 
